# Comparison of fetal growth patterns from Western India with Intergrowth-21^st^

**DOI:** 10.1371/journal.pone.0310710

**Published:** 2024-10-14

**Authors:** Arun Kinare, Priscilla Joshi, Kamini Dangat, Sanjay Gupte, Manish Tipnis, Garima Singh, Karuna Randhir, Shweta Madiwale, Hemlata Pisal, Girija Wagh, Sanjay Lalwani, Sadhana Joshi, Caroline Fall, Harshpal Singh Sachdev

**Affiliations:** 1 Department of Radiodiagnosis, Bharati Medical College and Hospital, Bharati Vidyapeeth (Deemed to be University), Pune, Maharashtra, India; 2 Mother and Child Health, ICMR–Collaborating Centre of Excellence (CCoE), Interactive Research School for Health Affairs, Bharati Vidyapeeth (Deemed to be University), Pune, Maharashtra, India; 3 Gupte Hospital and Research Centre, Pune, Maharashtra, India; 4 Department of Obstetrics and Gynaecology, Bharati Medical College and Hospital, Bharati Vidyapeeth (Deemed to be University), Pune, Maharashtra, India; 5 Department of Pediatrics, Bharati Medical College and Hospital, Bharati Vidyapeeth (Deemed to be University), Pune, Maharashtra, India; 6 MRC Lifecourse Epidemiology Centre, University of Southampton, Southampton, United Kingdom; 7 Sitaram Bhartia Institute of Science and Research, New Delhi, India; Hospital Sant Joan de Déu, SPAIN

## Abstract

**Objective:**

To generate longitudinal fetal growth data in an Indian population and compare it with Intergrowth-21^st^.

**Material and methods:**

Fetal biometry data was collected in a prospective longitudinal observational study (REVAMP: Research Exploring Various Aspects and Mechanisms in Preeclampsia) from 2017 to 2022. Fetal crown-rump length (CRL) was measured at 11–14 weeks gestation, and biparietal diameter (BPD), head circumference (HC), abdominal circumference (AC), and femur length (FL) at 18–22 and 32–35 weeks, and converted into Z-scores using the Intergrowth standard. Generalized Additive Models for Location, Scale and Shape (GAMLSS) models were used to construct fetal growth centile curves compared against Intergrowth centiles.

**Results:**

Out of 1096 singleton pregnancies in REVAMP, this analysis included 655 ‘healthy’ pregnancies (uncomplicated by pre-eclampsia, diabetes, pre-term delivery or low birth weight) and a sub-set of 106 ‘low-risk’ pregnancies defined using Intergrowth criteria. The ‘healthy’ study subjects showed lower mean CRL Z-score [-0.45 SD (95% CI:-0.54,-0.37)] at 11–14 weeks, and BPD Z-score [-1.2 SD (-1.28,-1.11) and -1.17 SD (-1.23,-1.1)] at 18–22 and 32–35 weeks respectively. Mean HC Z-score was comparable to the Intergrowth standard at 18–22 weeks [-0.08 SD (-0.16, 0.02)] but smaller at 32–35 weeks [-0.25 SD (-0.32,-0.19)]. Mean AC Z-score was lower at 18–22 weeks [-0.32 SD (-0.41,-0.23)] but comparable at 32–35 weeks [0.004 SD (-0.07, 0.07)]. FL was comparable to or larger than the Intergrowth standard at both time points [0.05 SD (-0.05, 0.14); 0.82 SD (0.75, 0.89), respectively]. These findings were similar, though measurements were slightly larger, in the ‘low-risk’ sample.

**Conclusions:**

This data from healthy and low-risk pregnant women in urban western India indicates that some fetal dimensions and growth trajectories differ significantly from the Intergrowth-21^st^. Our data suggest the need for a larger representative study to define a population-specific fetal growth reference for India, for identification of fetal growth restriction.

## Introduction

Intrauterine growth is a crucial indicator of a healthy pregnancy, and predicts future health prospects of the offspring [1, 2]. Suboptimal fetal growth significantly contributes to adverse perinatal health outcomes, including increased rates of morbidity and mortality [3, 4], and is also linked to heightened susceptibility to cardiovascular disease and diabetes mellitus later in adulthood [4, 5]. Therefore, early diagnosis of abnormal fetal growth or serial monitoring of fetal growth *in utero* is important for minimizing adverse short- and long-term health consequences [6–8]. Ultrasonographic measurements of fetal crown-rump length (CRL), biparietal diameter (BPD), femur length (FL), head circumference (HC), and abdominal circumference (AC) are used for assess fetal growth and accurately date pregnancies [9].

Fetal growth charts serve as tools for comparing the size of a fetus, whose gestational age is known, with reference data [10, 11]. Across the last four decades, multiple fetal growth charts have been formulated and refined, varying in study design, sample size, population, and statistical modeling methods [10, 11]. Three longitudinal growth charts have been established: the Fetal Growth Standard from the Eunice Kennedy Shriver National Institute of Child Health and Human Development (NICHD), the Fetal Growth Standard from the International Fetal and Newborn Growth Consortium for the 21^st^ Century (Intergrowth-21^st^), and the Fetal Growth Charts from the World Health Organization (WHO) [9, 12–14]. These studies included healthy women, but varied in their racial/ethnic background, socioeconomic and nutritional background and geographical location.

Most clinicians in developing countries like India use fetal growth charts that were developed in western populations [15–17]. However, these may not be applicable to the local population. It has been suggested that these growth charts may not be suitable for Asian newborns, who tend to be smaller than the population of high-income countries used to construct these references [16]. The Intergrowth-21^st^ study, based on a five year prospective study conducted in eight countries, including India, has been projected as the first global fetal and newborn growth standard, applicable in all populations. It is based on healthy urban women at lower risk of adverse maternal and perinatal outcomes [18, 19]. Despite extensive debate about its universal applicability, the Intergrowth standard is being increasingly used worldwide for interpreting routine ultrasound measurements [20, 21]. However, population-specific growth curves may be more useful for accurate fetal assessment. We report fetal growth data from a low-risk population in urban Pune, India and compare it with the standards established by the Intergrowth-21^st^.

## Material and methods

### Study site and participant selection

The study was undertaken in Pune, an urban area of Western India, from 2017–2022 as a part of the Indian Council of Medical Research (ICMR)-Centre for Advanced Research entitled “Investigating mechanisms leading to pre-eclampsia”(5/7/1069/13-RCH dated 31-03-2017). Ethics approval was obtained from the Bharati Vidyapeeth Institutional Ethics Committee, Pune (IEC/2015/37). Prior to data collection, each participant consented in writing. The REVAMP study, Research Exploring Various Aspects and Mechanisms in Pre-eclampsia, established a pregnant women cohort, who were monitored from early stages pregnancy until delivery. Pregnant women were recruited from two hospitals in Pune: Bharati Hospital, a large multi-specialty hospital catering mainly to middle-class and lower-middle-class patients and Gupte Hospital, a private hospital catering to more affluent patients and attracting many women with a poor obstetric history. The main purpose of this longitudinal study was to investigate the correlation between maternal long- chain polyunsaturated fatty acids and micronutrients with clinical outcome in pre-eclampsia. The study protocol has been published [22].

All pregnant women aged 18 to 45 years, of 11–14 weeks of gestation, attending the two hospitals for their routine antenatal care, planning to deliver at either hospital, and without major pre-existing chronic diseases (diabetes mellitus, heart disease, liver disorders, bleeding disorder, seizure disorder, and HIV/HBsAg infection) were enrolled in the study (n = 1,154). Women with multiple pregnancies, pregnancies in which a high nuchal translucency or, congenital malformations were detected, or abortion were excluded.

**Fig 1:**
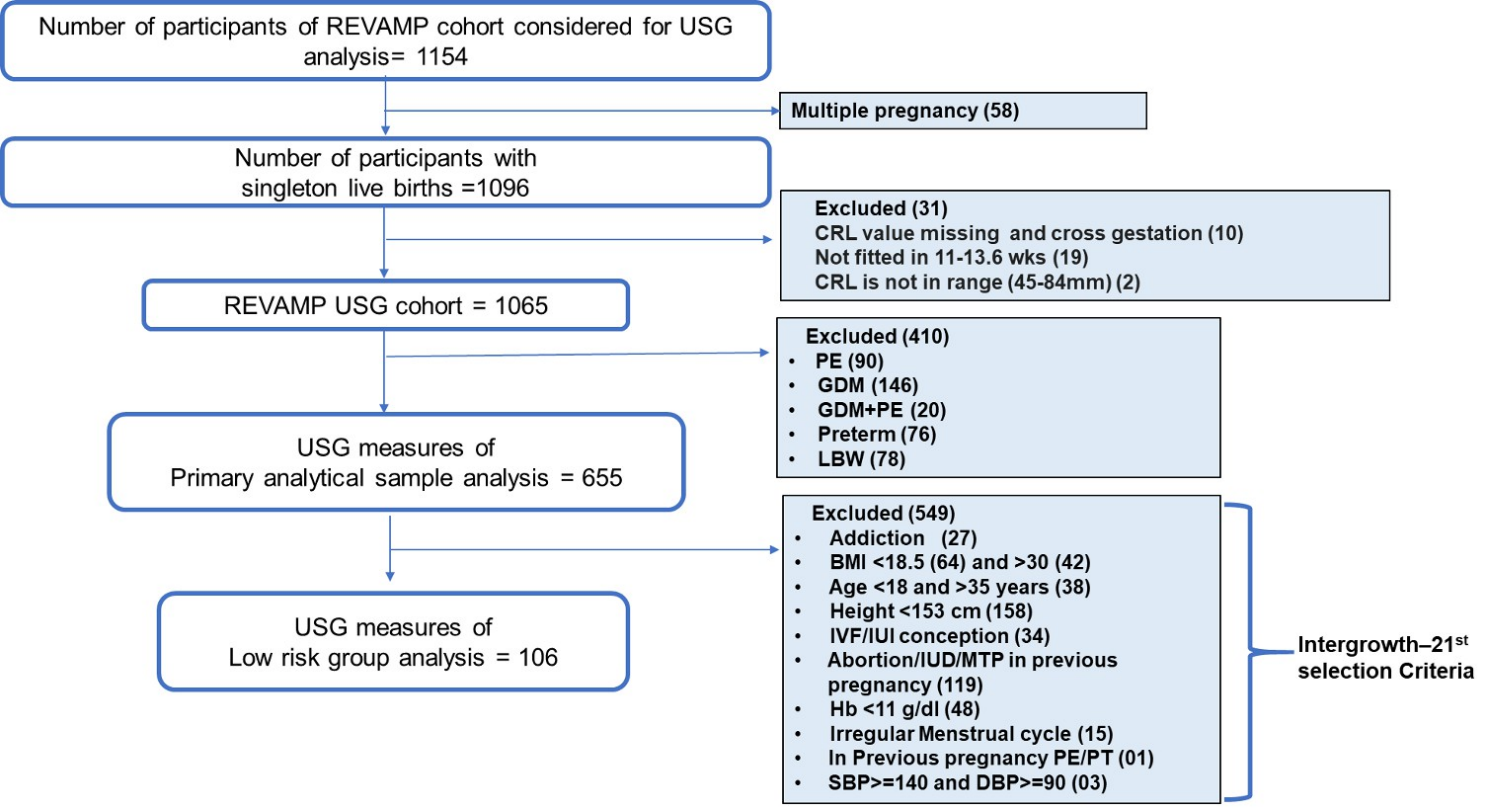
Flow chart of the study population. PE: preeclampsia; GDM: Gestational Diabetes Mellitus; LBW: Low Birth Weight; PT: pre-term; GWG: gestational weight gain; BMI: body mass index; IVF: *in vitro* fertilization; IUI: intrauterine insemination; Hb: haemoglobin; SBP: Systolic blood pressure; DBP: Diastolic blood pressure.

The recruitment period for this study was 27^th^ June 2017 to 17^th^ February 2020. 1154 women were enrolled in the REVAMP cohort, and were included in the current analysis. Of them, 58 women were excluded because of multiple pregnancy (leaving n = 1096). Information on ultrasound scan measures were not available for 31 women. The remaining sample was 1,065, of whom 524 were from Bharti Hospital and 541 from Gupte Hospital. We restricted the primary analysis to ‘healthy’ pregnancies by excluding women with negative pregnancy outcomes such as pre-eclampsia (10%), gestational diabetes mellitus (GDM 16%), pre-term delivery (7%), and low birth weight (7%) (n = 410) resulting in a sample size of 655 (Bharati hospital = 381, Gupte hospital = 274). We also classified a ’low-risk’ population, similar to the inclusion criteria of Intergrowth-21^st^ (S1 Table), which accounted for only 15.9% (106/655) participants in this cohort (Fig 1). Low birth weight for the purpose of this analysis was defined as babies weighing <2,500g.

### Data collection

#### Socio-demographic and clinical data

Socioeconomic and demographic data including family history and socioeconomic status; menstrual, obstetric, and clinical history; antenatal complications, and delivery details of the present pregnancy were recorded. The socioeconomic status was assessed using the Standard of Living Index, a measurement developed by the International Institute for Population Sciences, Mumbai and used in India’s National Family Health Survey 2 [23]. Occupational levels were categorized into professional, semi-professional, skilled worker and semi-skilled worker. Professionals include doctors, lawyers, engineers, professors, teachers, accountants, and police officers. In each trimester of pregnancy, anthropometric measures (height, weight), and periconceptional lifestyle including smoking, alcohol use, folic acid or multivitamin supplement intake), fetal ultrasonography, and color Doppler details were noted. Maternal weight (kg) was measured at 11–14, 18–22, 26–28 weeks, and at delivery, while height (cm) was measured once at the time of enrolment using a calibrated digital weighing scales and stadiometer.

Gestational age was assessed based on the last menstrual period (LMP) date. However, if there was a difference of more than ± 7 days between the gestational age calculated from the crown-rump length (CRL) measured during the initial ultrasound scan (at 11–14 weeks) and the LMP-based estimation, the CRL-derived gestational age was utilized. We have also calculated gestational age at delivery using both the LMP and CRL. Our findings indicate no significant differences in gestational age between the two methods (LMP: 39.17 weeks, CRL: 39.16 weeks). Data on birth outcomes were also recorded (date of birth, gender, birth weight, length,). Birth outcome measurements were recorded within 24 hour after birth. These measurements were taken by trained paediatrician following standardized protocols.

### Fetal biometry throughout pregnancy

All women were examined by ultrasound at enrolment at 11 to 14 weeks of gestational age to assess the CRL and to provide an ultrasound calculated gestation age. Further ultrasound examinations were carried out at 18–22 weeks and 32–35 weeks of gestation. All measurements were made following the protocols outlined by the International Society of Ultrasound in Obstetrics and Gynecology (ISCOG) and FMF guidelines [24]. Ultrasound scans were carried out by one of two trained sonologists, using GE VolusonE6 equipment (Wipro GE; General Electric Healthcare, Chicago, Illinois).

Fetal biometry measurements were performed using standard convex probes C1-6. CRL was measured at 11–14 weeks, and BPD, HC, AC, and FL at subsequent visits. BPD was measured from the outer proximal skull (nearest to the probe) to the inner distal skull (nearest to the probe). HC was the ovoid measurement of the entire skull bones at the level of the BPD, and the measurement was taken by following the ellipse curve along the outer edge of the skull. AC was measured at the level of the umbilical portion of the left portal vein using the ellipse curve. FL was measured from end to end with a full femoral image. Inter-observer variation analysis for all the ultrasound measurements was performed and the coefficients of variation were all <10%. The recorded values were compared with the reference values from Intergrowth-21^st^ for each parameter.

### Statistical analysis

Continuous variables including baseline characteristics such as socio-demographic and clinical were assessed within the study population and are presented as mean ± SD and/or 95% confidence intervals (CIs), and categorical variables as number (n) and percentage (%). The ultrasound measurements were converted into gestation-specific Z-scores and 95% CIs based on the Intergrowth-21^st^. Sonographic measurements were utilized to develop fetal growth charts for the ultrasound biometric parameters. (CRL, BPD, HC, AC, and FL). We constructed the 3^rd^, 10^th^, 25^th^, 50^th^, 75^th^, 90^th^, and 97^th^ percentiles of biometric parameters using the Lambda-Mu-Sigma (LMS) method via GAMLSS models (Generalized Additive Models for Location, Scale and Shape package; version 5.1–6) in the R software (version 4.1.2) [25]. We plotted and compared these centiles with the Intergrowth-21^st^ [26].

## Results

### Maternal socio-demographic characteristics and birth outcomes

Table 1 displays maternal characteristics and birth outcomes. The mean maternal age was 28 years, and mean body mass index (BMI) was 23.4 kg/m^2^. One-quarter (27.1%) of women were anaemic, 38% were primigravida, and 69.4% were of graduate or higher educational status. The average gestational age at delivery was 39.0 weeks with a standard deviation of 0.94 weeks and 53.6% women delivered by caesarean section. The average birth weight and length were 2952 ± 334 grams and 48.8 ± 3.8 cm, respectively (Table 1).

**Table 1:** Socio-demographic and clinical characteristics of the primary analytical sample and the low-risk pregnant women.

Characteristics	Primary analytical data	Low risk population
	(n = 655)	(n = 106)
**Age (yrs)**	28.0 ± 4.5	26.5 ± 4.1
**BMI (kg/m²)**	23.4 ± 4.2	22.9 ± 2.9
**Education n (%)**		
***Graduates and Above***	452 (69.4)	72 (67.9)
**Occupation n (%)**		
***Professional***	202 (31.2)	29 (27.4)
**Gravida n (%)**		
***Primigravida***	247 (37.7)	66 (62.3)
**Parity n (%)**		
***Nulliparous***	374 (57.1)	66 (62.3)
**Caesarean section delivery n (%)**	351(53.6)	45 (42.4)
**Gestational age at birth (weeks)**	39.0 ± 0.94	39.1 ± 0.93
**Baby Gender**		
**Male n (%)**	301 (45.9)	43 (40.6)
**Female n (%)**	354 (54.1)	63 (59.4)
**Birth weight (gms)**	2952.2 ± 334.6	2953.4 ± 361.1
**Birth length (cm)**	48.8 ± 3.8	49.2 ± 3.7

Values are expressed as Mean ± SD. BMI: Body Mass Index; SLI: Standard of Living Index; n: Number of subjects HC: Head Circumference; CC: Chest circumference; In primary analytical data and low risk population Birth weight n = 655 and 640 respectively. Similarly, birth length in primary analytical data and low risk population n = 106 and n-102 respectively

### Comparison of REVAMP fetal growth pattern to Intergrowth-21^st^

**Fig 2:**
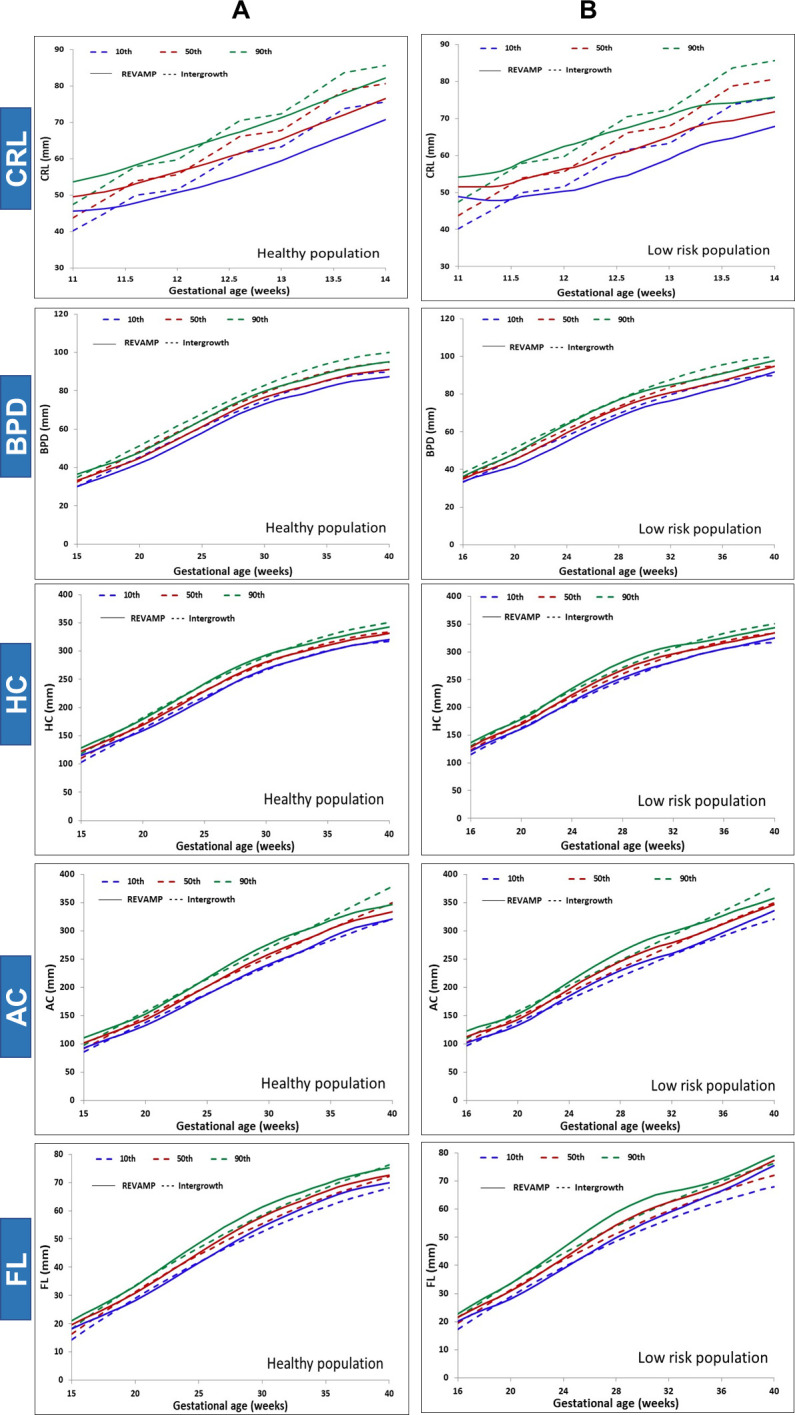
Graphic comparison of the REVAMP fetal growth percentiles with INTERGROWTH-21^st^ (A) Full healthy population (B) Low-risk population. CRL: Crown-rump length; BPD: biparietal diameter; HC: head circumference; AC: abdominal circumference; FL: femur length.

Mean gestational age for the three ultrasound scans during pregnancy were 12.3, 18.6, and 34.4 weeks. Table 2 shows the mean (SD, 95% CI) values for the ultrasound measurements and their Z-scores, and Fig 2 show the data in graphic form as growth curves. All fetal biometry Z-scores had a Gaussian distribution.

**Table 2:** Z-scores for CRL, BPD, HC, AC, and FL in comparison with Intergrowth-21^st^.

Time points	Mean Gestation (weeks)		CRL	BPD	HC	AC	FL
**Total population**							
**11–14 weeks (n = 655)**	**12.3**	**Mean (mm)**	**60.1**	**NA**	**NA**	**NA**	**NA**
		**Z-scores**	**-0.45 ± 1.13**				
		**95% CI**	**(-0.54,-0.37)**				
**18–22 weeks (n = 639)**	**18.6**	**Mean (mm)**	**NA**	**42.2**	**158.2**	**133.0**	**28.2**
		**Z-scores**		**-1.2 ± 1.07**	**-0.08 ± 1.10**	**-0.32 ± 1.14**	**0.05 ± 1.12**
		**95% CI**		**(-1.28,-1.11)**	**(-0.16, 0.02)**	**(-0.41,-0.23)**	**(-0.05,0.14)**
**32–35 weeks (n = 635)**	**34.4**	**Mean (mm)**	**NA**	**84.7**	**308.2**	**299.4**	**66.1**
		**Z-scores**		**-1.17 ± 0.87**	**-0.25 ± 0.84**	**0.004 ± 0.85**	**0.82 ± 0.90**
		**95% CI**		**(-1.23,-1.10)**	**(-0.32,-0.19)**	**(-0.07,0.07)**	**(0.75,0.89)**
**Low risk population**							
**11–14 weeks (n = 106)**	**12.2**	**Mean (mm)**	**59.9**	**NA**	**NA**	**NA**	**NA**
		**Z-score**	**-0.38 ± 1.17**				
		**95% CI**	**(-0.59, -0.15)**				
**18–22 weeks (n = 105)**	**18.6**	**Mean (mm)**	**NA**	**42.4**	**159.5**	**133.9**	**28.4**
		**Z-scores**		**-1.11 ± 1.03**	**0.10 ± 0.98**	**-0.21 ± 1.21**	**0.17 ± 1.12**
		**95% CI**		**(-1.29, -0.90)**	**(-0.10,0.28)**	**(-0.44,0.03)**	**(-0.06,0.37)**
**32–35 weeks (n = 104)**	**34.4**	**Mean (mm)**	**NA**	**85.0**	**309.3**	**300.4**	**66.5**
		**Z-scores**		**-1.1 ± 0.93**	**-0.15 ± 0.94**	**0.08 ± 0.91**	**0.98 ± 0.9**
		**95% CI**		**(-1.29, -0.92)**	**(-0.33,0.05)**	**(-0.11,0.25)**	**(0.81,1.16)**

Data expressed as Actual Mean, Z-scores (95% confidence intervals); CRL: Crown-rump length; BPD: biparietal diameter; HC: head circumference; AC: abdominal circumference

FL: femur length; SDS, standard deviation score; NA: Not applicable

CRL was significantly smaller in the REVAMP cohort than the Intergrowth, with mean Z-scores [-0.45 SD (95% CI: -0.54, -0.37)] at 11–14 weeks of in the full (‘healthy’) sample and -0.38 SD (-0.59, -0.15) in the ‘low-risk’ subset (Table 2). In both samples the REVAMP fetuses tended to have higher centiles at 11–12 weeks gestation relative to the Intergrowth than after 12 weeks as shown in Fig 2A and 2B (S2 Table).

At 18–22 weeks and 32–35 weeks, mean BPD Z-scores were lower than the Intergrowth-21^st^ in both the total and low risk group (Table 2). These differences were clinically important, all around one standard deviation, and were consistent throughout pregnancy in both groups (Fig 2A and 2B and S3 Table). HC Z-scores were much closer than BPD measurements to the Intergrowth, and all deficits were less than 0.3 SD in magnitude (Table 2). Mean Z-scores were comparable to the Intergrowth at 18–22 weeks in both samples, but significantly lower, by 0.25 SD, at 32–35 weeks in the total population (Fig 2A and 2B and S4 Table). AC Z-scores were lower than the Intergrowth at 18–22 weeks in both groups by 0.2–0.3 SD (Table 2). At 32–35 weeks, however, they were comparable to Intergrowth (Fig 2A and 2B and S5 Table). FL showed a different growth pattern again. Mean Z-scores were higher than the Intergrowth in both the second and third trimesters, significantly higher (0.8–0.9 SD) in the third trimester (Table 2 and Fig 2A and 2B and S6 Table).

These results are mirrored in the data for the percentages of fetuses with measurements below the 10^th^ percentile and above the 90^th^ percentile (Table 3). At 11–14 weeks the percentages of fetuses with CRL measurements below the 10^th^ percentile were similar in the full healthy sample (23.4%) and the low-risk group (22.7%). When GA was calculated based on CRL, we found that percentages of fetuses with CRL measurements were similar for both methods. It was 24.5% below the 10^th^ centile in the low risk population. High percentages of fetuses in both groups had BPD measurements below the 10^th^ percentile throughout pregnancy (38–48%). These percentages were much lower for HC, AC and FL, and all less than 10% in the final trimester (only 1% for FL). Over 20% of fetuses had FL measurements above the 90^th^ Intergrowth percentile in the last trimester. Throughout pregnancy, fetuses were larger in the low-risk than in the full healthy sample. The birth measurements showed similar percentages of babies in both groups (13–15% for birth weight and 16–19% for birth length) were below the 10^th^ Intergrowth percentile. However, while only 2–3% were above the 90^th^ percentile for birth weight, 26–28% were above the 90^th^ percentile for birth length (Table 3).

**Table 3:** Percentage of fetuses under the 10^th^ centile or above the 90^th^ centile using Intergrowth-21^st^ charts.

Weeks of Gestation	Group	Less than 10^th^ centile % (95% CI)	More than 90^th^ centile % (95% CI)
	**CRL**		
**11–14 weeks**	Total	23.4 (20.1, 26.7)	7.1 (5.2, 9.0)
	Low risk	22.7 (15.4, 30.7)	10.4 (4.9, 17.3)
	**BPD**		
**18–22 weeks**	Total	48 (44.1, 52.3)	1.3 (0.5, 2.2)
	Low risk	42.4 (32.7, 51.9)	1 (0, 3.4)
**32–35 weeks**	Total	43.1 (39.1, 47.0)	0.2 (0, 0.5)
	Low risk	38.5 (29.4, 47.9)	1 (0, 3.6)
	**HC**		
**18–22 weeks**	Total	11.4 (8.9, 14.0)	8.1 (6.1, 10.3)
	Low risk	8.7 (3.9, 14.8)	9.7 (4.4, 15.5)
**32–35 weeks**	Total	9.6 (7.5, 11.7)	3.6 (2.2, 5.1)
	Low risk	7.7 (3.1, 13.2)	6.8 (2.2, 12.3)
	**AC**		
**18–22 weeks**	Total	17.4 (14.6, 20.3)	6.2 (4.3, 8.2)
	Low risk	19.7 (11.5, 27.6)	7.9 (3.0, 13.0)
**32–35 weeks**	Total	5.2 (3.5, 7.1)	5.5 (3.8, 7.4)
	Low risk	2.9 (0, 6.6)	6.8 (2.2, 11.8)
	**FL**		
**18–22 weeks**	Total	11.4 (8.9, 14.0)	11.5 (9.2, 14.1)
	Low risk	13.5 (7.2, 20.0)	14.5 (8.1,21.5)
**32–35 weeks**	Total	1 (0.3,1.8)	26.5 (23.0, 29.9)
	Low risk	1 (0, 3.2)	28.2 (19.7, 36.4)

Crown-rump length; BPD: biparietal diameter; HC: head circumference; AC: abdominal circumference; FL: femur length

## Discussion

The current study describes ultrasound measurements of fetal growth from 11–40 weeks among healthy pregnant women recruited from two urban hospitals in Pune, Western India. In this prospective cohort study we compared fetal CRL, BPD, HC, AC and FL with the Intergrowth-21^st^ [12], including a comparison within a low-risk sub-set of women selected to match the Intergrowth recruitment criteria. Even in the low-risk group, there were significant differences in fetal size between our study and the Intergrowth, and these differences varied according to the fetal measurement and gestational stage. Notably 1^st^ trimester CRL was approximately 0.4 SD smaller, and BPD in the 2^nd^ and 3^rd^ trimesters was approximately 1 SD smaller than the Intergrowth. HC was comparable with the Intergrowth at 18–22 weeks, but faltered in late gestation to become approximately 0.2 SD smaller. In contrast, AC was smaller at 18–22 weeks, but caught up to become comparable to the Intergrowth in late gestation. FL was larger than the Intergrowth throughout gestation, especially in the last trimester, achieving values nearly 1 SD higher than the Intergrowth, even in the full sample. In summary, our results suggest that Indian fetuses have markedly smaller CRL in early pregnancy and BPD throughout pregnancy than the Intergrowth reference, but relatively preserved limb length and abdominal circumference. Fetal measurements were always higher in the full sample than in the low-risk group, highlighting the importance for fetal growth of maternal age, stature, overall health, optimal body weight, and absence of risk factors such as tobacco use. It was striking that a very large proportion of our study sample (84%) were excluded under the Intergrowth criteria for a low-risk pregnancy.

The Intergrowth-21^st^ used data from eight countries, including India, to provide a standardized fetal growth measurement reference chart. The participants from India in Intergrowth-21^st^ were from Nagpur, the same state as Pune. The Intergrowth-21^st^ study concluded that despite the diverse geographical settings, fetal growth is similar all over the world among low-risk healthy women. However, it is mentioned that in some of the study populations, including India, fetal head circumference was in the lower range compared to other countries [18]. Subsequently, another study conducted among non-affluent healthy women from Northern India (Delhi), also reported smaller fetal size compared to the Intergrowth-21^st^ [27]. This raised questions about the routine use of the Intergrowth standard for monitoring fetal growth in India, because of the potential risk of over-diagnosing fetal growth restriction.

There have been several studies of fetal biometry in India, mostly showing, like our study, smaller fetal size in early gestation than other standards. Two studies from the Punjab and New Delhi in India reported smaller fetal biometric measures at 18–28 weeks and 29–38 weeks [28] and at 5 time points ranging from 10–40 weeks [27] of gestation as compared to Hadlock’s reference values and the Intergrowth-21^st^, respectively. Another study from Pune India, reported that all measurements (BPD, HC, AC) except FL were smaller for Indian fetuses at 18, 30 and 36 weeks compared with a European reference [16]. The Mumbai Maternal Nutrition Project (MMNP) among pregnant women living in urban slums, reported that fetuses had significantly smaller CRL at 9–12 weeks and head and abdominal circumferences at 28–32 weeks compared with the Intergrowth-21^st^ [29]. In contrast, a study from Gujarat, India concluded that BPD, HC and FL were comparable to western standards (Hadlock, Campbell, Jeanty and Chitty) up to 34 weeks gestation and AC was comparable until the end of the 30^th^ week [30]. Most of these studies did not specifically analyse a low-risk sub-group. However, our data suggest that more work may be needed to determine whether the Intergrowth reference is truly applicable to India or whether a population-specific reference is indicated. A much broader sample, and larger sample size, than ours would be required for this.

The relative preservation of femur length seen in our study throughout gestation is interesting. Ruvolo et al. assessed a racially diverse population consisting of Blacks, Asians, and Caucasians in the USA found no variation in FL between these populations [31]. Kinare et al (2010) reported that while other ultrasound measurements were smaller, FL in a rural Indian population was comparable to a European reference [16], and di Gravio et al (2019) reported that median fetal FL was almost identical to the Intergrowth median in the 2^nd^ and 3^rd^ trimesters [29].

A striking finding in our study was the large number of women (410, 38%) who had to be excluded from our analysis sample because of pregnancy complications, including GDM, pre-eclampsia, pre-term delivery or low birth weight. Similarly, a further 549 were excluded to derive the low-risk sample defined using the Intergrowth criteria, the main reasons being short stature, low or high BMI, anaemia, extremes of maternal age, and assisted conception. This may be partly explained by the fact that the Gupte Hospital attracts a disproportionate number of women with an adverse obstetric history. However, it attests to high levels of sub-optimal maternal weight and height, and of gestational morbidities such as GDM and pre-eclampsia. The high incidence of GDM and pre-eclampsia is consistent with other Indian studies and is a cause for concern. The fact that fetal size was larger among women in the low-risk group is consistent with the known associations of maternal age, BMI, height, socio-economic status, nutritional adequacy and multiple environmental factors with fetal growth [29, 32, 33].

The strengths of the present study were standardised fetal ultrasound measurements recorded by two experienced ultrasonologists at 3 time points (between 11 to 40 weeks of gestation) in a sample of very well-characterised women. A limitation was the small sample size in the low-risk group. Our study included women residing in an urban area with relatively robust access to healthcare, and we did not include rural women.

## Conclusion

This study describes fetal growth data from ‘healthy’ pregnant women in urban western India, and indicates that some fetal growth dimensions (CRL, BPD, and HC) are significantly smaller than the Intergrowth-21^st^. In contrast, FL was similar or larger. AC was lower in early pregnancy but was similar to the Intergrowth reference values in the latter half of pregnancy. Additional multicentric longitudinal data will help in developing national guidelines and better evaluation of fetal growth.

## Supporting information

S1 Table Intergrowth-21^st^ exclusion criteria. (DOCX)

S2 Table Comparison of REVAMP cohort CRL centiles with Intergrowth-21^st^ centiles. CRL: Crown-rump length.
(DOCX)

S3 Table Comparison of REVAMP cohort BPD centiles with Intergrowth-21^st^ centiles. BPD: biparietal diameter.
(DOCX)

S4 Table Comparison of REVAMP cohort HC centiles with Intergrowth-21^st^ centiles. HC: head circumference.
(DOCX)

S5 Table Comparison of REVAMP cohort AC centiles with Intergrowth-21^st^ centiles. AC: abdominal circumference.
(DOCX)

S6 Table Comparison of REVAMP cohort FL centiles with Intergrowth-21^st^ centiles. FL: femur length.
(DOCX)

## Abbreviations

CRL: crown-rump length
BPD: biparietal diameter
FL: femur length
HC: head circumference
AC: abdominal circumference
