# Supplementary material for: Comparison of fetal growth patterns from Western India with Intergrowth-21st
Source: PLoS One. 2024 Oct 14;19(10):e0310710. doi: 10.1371/journal.pone.0310710 (PMC11472910; doi:10.1371/journal.pone.0310710)
Supplement: S1 Table — (DOCX) [file pone.0310710.s001.docx]

**S1 Table: Intergrowth-21^st^ exclusion criteria**

| - Aged ≥18 and <35 years |
| --- |
| - Body mass index ≥18.5 and <30 kg/m^3^ |
| - Height ≥153 cm |
| - Singleton pregnancy |
| - A known last menstrual period with regular cycles (defined as 28 ± 4 days) without hormonal contraceptive use, or breastfeeding in the months before pregnancy |
| - Natural conception |
| - No relevant past medical history, with no need for long-term medication (including fertility treatment and over-the-counter medicines, but excluding routine iron, folate, calcium, iodine or multivitamin supplements). |
| - No evidence of socioeconomic constraints likely to impede fetal growth identified using local definitions of social risk |
| - No use of tobacco or recreational drugs such as cannabis in the 3 months before or after becoming pregnant. |
| - No heavy alcohol use (defined as >5 units - (50 ml pure alcohol) per week) since becoming pregnant. |
| - No more than one miscarriage in the two previous consecutive pregnancies. |
| - No previous baby delivered preterm (<37+0 weeks of gestation) or with a birth weight <2500 g or >4500 g. |
| - No previous neonatal or fetal death, previous baby with any congenital malformations, and no evidence in present pregnancy of congenital disease or fetal anomaly. |
| - No previous pregnancy affected by pre-eclampsia/eclampsia, HELLP syndrome or a related pregnancy-associated condition. |
| - No clinically significant atypical red cell alloantibodies. |
| - Negative urinalysis |
| - Systolic blood pressure <140 mmHg and diastolic blood pressure <90 mmHg. |
| - No diagnosis or treatment for anaemia during this pregnancy (haemoglobin levels will be monitored throughout pregnancy). |
| - No clinical evidence of any other sexually transmitted diseases, including syphilis and clinical trichomoniasis. |
| - Not in an occupation with risk of exposure to chemicals or toxic substances, or very physically demanding activity to be evaluated by local standards. Also women should not be conducting vigorous or contact sports, such as scuba diving or similar activities. |
