# Supplementary material for: Comparison of fetal growth patterns from Western India with Intergrowth-21st
Source: PLoS One. 2024 Oct 14;19(10):e0310710. doi: 10.1371/journal.pone.0310710 (PMC11472910; doi:10.1371/journal.pone.0310710)
Supplement: S2 Table — CRL: Crown-rump length. (DOCX) [file pone.0310710.s002.docx]

**S2 Table: Comparison of REVAMP cohort CRL centiles with Intergrowth-21^st^ centiles**

| **CRL** | **Intergrowth 21^st^** | | | **REVAMP cohort**  **Total population**  **(655)** | | | **REVAMP cohort**  **Low risk population (106)** | | |
| --- | --- | --- | --- | --- | --- | --- | --- | --- | --- |
|  | **10^th^** | **50^th^** | **90^th^** | **10^th^** | **50^th^** | **90^th^** | **10^th^** | **50^th^** | **90^th^** |
| 11+0 | 40.2 | 43.8 | 47.5 | 45.6 | 49.6 | 53.6 | 48.8 | 51.5 | 54.2 |
| 11+1 | 41.8 | 45.5 | 49.2 | 45.8 | 50.0 | 54.3 | 48.5 | 51.5 | 54.5 |
| 11+2 | 43.4 | 47.1 | 50.9 | 46.0 | 50.4 | 54.9 | 48.1 | 51.5 | 54.8 |
| 11+3 | 45.0 | 48.8 | 52.6 | 46.2 | 50.9 | 55.6 | 47.8 | 51.5 | 55.2 |
| 11+4 | 46.6 | 50.5 | 54.4 | 46.6 | 51.5 | 56.5 | 47.8 | 51.8 | 55.8 |
| 11+5 | 48.2 | 52.2 | 56.1 | 47.2 | 52.3 | 57.3 | 48.1 | 52.5 | 56.8 |
| 11+6 | 49.9 | 53.9 | 57.9 | 47.8 | 53.1 | 58.3 | 48.9 | 53.5 | 58.2 |
| 12+0 | 51.5 | 55.6 | 59.7 | 50.7 | 56.4 | 62.1 | 50.3 | 56.3 | 62.4 |
| 12+1 | 53.2 | 57.3 | 61.5 | 51.4 | 57.2 | 63.0 | 50.6 | 56.8 | 63.1 |
| 12+2 | 54.8 | 59.0 | 63.3 | 52.1 | 58.0 | 63.9 | 51.3 | 57.7 | 64.0 |
| 12+3 | 56.5 | 60.8 | 65.1 | 52.9 | 58.8 | 64.8 | 52.3 | 58.7 | 65.1 |
| 12+4 | 58.2 | 62.5 | 66.9 | 53.7 | 59.7 | 65.7 | 53.3 | 59.7 | 66.1 |
| 12+5 | 59.9 | 64.3 | 68.7 | 54.6 | 60.6 | 66.5 | 54.0 | 60.4 | 66.9 |
| 12+6 | 61.6 | 66.1 | 70.5 | 55.5 | 61.5 | 67.4 | 54.6 | 61.0 | 67.5 |
| 13+0 | 63.3 | 67.8 | 72.4 | 59.5 | 65.4 | 71.2 | 59.0 | 65.0 | 70.9 |
| 13+1 | 65.0 | 69.6 | 74.3 | 60.6 | 66.5 | 72.4 | 60.4 | 66.2 | 71.9 |
| 13+2 | 66.8 | 71.4 | 76.1 | 61.8 | 67.7 | 73.6 | 61.7 | 67.3 | 72.8 |
| 13+3 | 68.5 | 73.3 | 78.0 | 63.0 | 68.8 | 74.7 | 62.8 | 68.2 | 73.5 |
| 13+4 | 70.3 | 75.1 | 79.9 | 64.1 | 70.0 | 75.8 | 63.6 | 68.7 | 73.9 |
| 13+5 | 72.0 | 76.9 | 81.8 | 65.2 | 71.1 | 76.9 | 64.2 | 69.1 | 74.1 |
| 13+6 | 73.8 | 78.8 | 83.7 | 66.3 | 72.1 | 78.0 | 64.7 | 69.5 | 74.2 |
| 14+0 | 75.6 | 80.6 | 85.7 | 70.8 | 76.5 | 82.3 | 67.8 | 71.8 | 75.7 |

CRL: Crown rump-length
