# Supplementary material for: Comparison of fetal growth patterns from Western India with Intergrowth-21st
Source: PLoS One. 2024 Oct 14;19(10):e0310710. doi: 10.1371/journal.pone.0310710 (PMC11472910; doi:10.1371/journal.pone.0310710)
Supplement: S3 Table — BPD: biparietal diameter. (DOCX) [file pone.0310710.s003.docx]

**S3 Table: Comparison of REVAMP cohort BPD centiles with Intergrowth-21^st^ centiles**

| **BPD** | **Intergrowth 21^st^** | | | **REVAMP cohort**  **Total population**  **(655)** | | | **REVAMP cohort**  **Low risk population (106)** | | |
| --- | --- | --- | --- | --- | --- | --- | --- | --- | --- |
|  | **10^th^** | **50^th^** | **90^th^** | **10^th^** | **50^th^** | **90^th^** | **10^th^** | **50^th^** | **90^th^** |
| 14 | 27.4 | 29.6 | 31.8 |  |  |  |  |  |  |
| 15 | 30.2 | 32.6 | 34.9 | 30.0 | 33.3 | 36.6 | 31.0 | 32.0 | 33.1 |
| 16 | 33.2 | 35.7 | 38.1 | 32.4 | 35.5 | 38.7 | 33.7 | 35.0 | 36.3 |
| 17 | 36.2 | 38.8 | 41.4 | 34.8 | 37.8 | 40.7 | 36.1 | 37.8 | 39.6 |
| 18 | 39.3 | 42.0 | 44.7 | 37.1 | 40.0 | 42.8 | 38.0 | 40.2 | 42.5 |
| 19 | 42.4 | 45.2 | 48.0 | 39.5 | 42.3 | 45.1 | 39.7 | 42.6 | 45.4 |
| 20 | 45.5 | 48.4 | 51.4 | 42.1 | 44.9 | 47.8 | 41.8 | 45.2 | 48.6 |
| 21 | 48.6 | 51.7 | 54.8 | 45.0 | 48.0 | 51.0 | 44.7 | 48.5 | 52.3 |
| 22 | 51.8 | 55.0 | 58.1 | 48.1 | 51.2 | 54.3 | 48.0 | 52.2 | 56.3 |
| 23 | 54.9 | 58.2 | 61.5 | 51.3 | 54.5 | 57.7 | 51.4 | 55.8 | 60.2 |
| 24 | 58.0 | 61.4 | 64.8 | 54.7 | 57.9 | 61.2 | 54.9 | 59.4 | 64.0 |
| 25 | 61.0 | 64.5 | 68.0 | 58.1 | 61.4 | 64.6 | 58.4 | 63.0 | 67.6 |
| 26 | 64.0 | 67.6 | 71.2 | 61.5 | 64.8 | 68.1 | 61.7 | 66.3 | 70.9 |
| 27 | 66.9 | 70.6 | 74.3 | 64.8 | 68.1 | 71.4 | 65.0 | 69.5 | 74.1 |
| 28 | 69.7 | 73.5 | 77.3 | 67.9 | 71.2 | 74.5 | 68.0 | 72.5 | 76.9 |
| 29 | 72.4 | 76.3 | 80.1 | 70.8 | 74.0 | 77.3 | 70.7 | 75.1 | 79.5 |
| 30 | 75.0 | 78.9 | 82.8 | 73.2 | 76.5 | 79.9 | 73.1 | 77.4 | 81.7 |
| 31 | 77.4 | 81.4 | 85.4 | 75.2 | 78.6 | 82.0 | 75.0 | 79.3 | 83.5 |
| 32 | 79.7 | 83.8 | 87.8 | 76.8 | 80.3 | 83.8 | 76.6 | 80.8 | 85.0 |
| 33 | 81.8 | 85.9 | 90.1 | 78.3 | 81.9 | 85.5 | 78.1 | 82.2 | 86.2 |
| 34 | 83.7 | 87.9 | 92.2 | 80.0 | 83.7 | 87.3 | 80.0 | 83.9 | 87.7 |
| 35 | 85.3 | 89.7 | 94.0 | 81.9 | 85.5 | 89.2 | 81.9 | 85.6 | 89.2 |
| 36 | 86.8 | 91.2 | 95.7 | 83.5 | 87.2 | 90.9 | 83.6 | 87.3 | 90.9 |
| 37 | 88.0 | 92.5 | 97.1 | 84.9 | 88.6 | 92.3 | 85.6 | 89.1 | 92.7 |
| 38 | 88.9 | 93.6 | 98.3 | 85.8 | 89.6 | 93.3 | 87.6 | 91.0 | 94.4 |
| 39 | 89.6 | 94.4 | 99.2 | 86.5 | 90.4 | 94.3 | 89.7 | 92.9 | 96.2 |
| 40 | 89.9 | 94.9 | 99.9 | 87.2 | 91.2 | 95.2 | 91.7 | 94.8 | 97.9 |

BPD: Biparietal-diameter
