# Supplementary material for: Comparison of fetal growth patterns from Western India with Intergrowth-21st
Source: PLoS One. 2024 Oct 14;19(10):e0310710. doi: 10.1371/journal.pone.0310710 (PMC11472910; doi:10.1371/journal.pone.0310710)
Supplement: S4 Table — HC: head circumference. (DOCX) [file pone.0310710.s004.docx]

**S4 Table:** **Comparison of REVAMP cohort HC centiles with Intergrowth-21^st^ centiles**

| **HC** | **Intergrowth 21^st^** | | | **REVAMP cohort**  **Total population**  **(655)** | | | **REVAMP cohort**  **Low risk population (109)** | | |
| --- | --- | --- | --- | --- | --- | --- | --- | --- | --- |
|  | **10^th^** | **50^th^** | **90^th^** | **10^th^** | **50^th^** | **90^th^** | **10^th^** | **50^th^** | **90^th^** |
| 14 | 90.7 | 97.9 | 105.0 |  |  |  |  |  |  |
| 15 | 102.8 | 110.4 | 118.0 | 115.0 | 122.0 | 129.0 | 109.5 | 116.7 | 123.9 |
| 16 | 114.9 | 122.9 | 130.9 | 123.9 | 131.3 | 138.7 | 121.3 | 128.8 | 136.3 |
| 17 | 127.0 | 135.4 | 143.9 | 132.7 | 140.5 | 148.3 | 132.7 | 140.5 | 148.3 |
| 18 | 139.1 | 147.9 | 156.7 | 141.4 | 149.6 | 157.9 | 142.8 | 150.9 | 158.9 |
| 19 | 151.1 | 160.3 | 169.5 | 150.1 | 159.0 | 168.0 | 151.9 | 160.2 | 168.5 |
| 20 | 163.0 | 172.5 | 182.0 | 159.4 | 169.2 | 179.0 | 161.0 | 169.8 | 178.5 |
| 21 | 174.7 | 184.5 | 194.3 | 169.6 | 180.4 | 191.2 | 172.5 | 181.9 | 191.4 |
| 22 | 186.2 | 196.3 | 206.4 | 180.3 | 192.1 | 203.9 | 185.5 | 195.7 | 205.9 |
| 23 | 197.5 | 207.8 | 218.2 | 191.6 | 204.2 | 216.8 | 198.4 | 209.4 | 220.3 |
| 24 | 208.5 | 219.1 | 229.7 | 203.3 | 216.4 | 229.6 | 210.8 | 222.5 | 234.2 |
| 25 | 219.1 | 230.0 | 240.8 | 215.3 | 228.7 | 242.0 | 222.6 | 235.1 | 247.5 |
| 26 | 229.5 | 240.5 | 251.6 | 227.3 | 240.7 | 254.1 | 233.7 | 246.9 | 260.1 |
| 27 | 239.4 | 250.7 | 261.9 | 239.0 | 252.2 | 265.4 | 244.0 | 257.9 | 271.7 |
| 28 | 248.9 | 260.4 | 271.8 | 250.0 | 262.9 | 275.9 | 253.5 | 267.9 | 282.3 |
| 29 | 258.0 | 269.6 | 281.3 | 260.0 | 272.6 | 285.2 | 262.1 | 276.9 | 291.6 |
| 30 | 266.5 | 278.4 | 290.2 | 268.8 | 281.0 | 293.2 | 269.8 | 284.7 | 299.6 |
| 31 | 274.6 | 286.6 | 298.7 | 276.2 | 288.0 | 299.9 | 276.5 | 291.2 | 305.9 |
| 32 | 282.1 | 294.4 | 306.7 | 282.3 | 293.9 | 305.4 | 282.5 | 296.6 | 310.7 |
| 33 | 288.9 | 301.5 | 314.1 | 288.0 | 299.2 | 310.4 | 288.6 | 301.3 | 314.0 |
| 34 | 295.2 | 308.1 | 321.0 | 294.2 | 304.9 | 315.7 | 295.1 | 306.2 | 317.3 |
| 35 | 300.8 | 314.1 | 327.4 | 300.3 | 310.7 | 321.0 | 301.1 | 311.2 | 321.4 |
| 36 | 305.6 | 319.4 | 333.2 | 305.6 | 315.7 | 325.8 | 305.6 | 315.6 | 325.6 |
| 37 | 309.8 | 324.1 | 338.4 | 310.1 | 320.2 | 330.4 | 310.0 | 320.1 | 330.1 |
| 38 | 313.1 | 328.1 | 343.0 | 313.8 | 324.3 | 334.7 | 315.0 | 324.8 | 334.7 |
| 39 | 315.7 | 331.4 | 347.1 | 317.0 | 327.9 | 338.9 | 320.0 | 329.6 | 339.3 |
| 40 | 317.4 | 333.9 | 350.5 | 319.9 | 331.5 | 343.0 | 325.0 | 334.5 | 343.9 |

HC: Head circumference
