# Supplementary material for: Comparison of fetal growth patterns from Western India with Intergrowth-21st
Source: PLoS One. 2024 Oct 14;19(10):e0310710. doi: 10.1371/journal.pone.0310710 (PMC11472910; doi:10.1371/journal.pone.0310710)
Supplement: S5 Table — AC: abdominal circumference. (DOCX) [file pone.0310710.s005.docx]

**S5 Table: Comparison of REVAMP cohort AC centiles with Intergrowth-21^st^ centiles**

| **AC** | **Intergrowth 21^st^** | | | **REVAMP cohort**  **Total population**  **(655)** | | | **REVAMP cohort**  **Low risk population (106)** | | |
| --- | --- | --- | --- | --- | --- | --- | --- | --- | --- |
|  | **10^th^** | **50^th^** | **90^th^** | **10^th^** | **50^th^** | **90^th^** | **10^th^** | **50^th^** | **90^th^** |
| 14 | 75.3 | 80.6 | 85.9 |  |  |  |  |  |  |
| 15 | 85.8 | 91.9 | 98.1 | 93.0 | 102.0 | 110.9 | 93.8 | 104.1 | 114.3 |
| 16 | 96.3 | 103.2 | 110.1 | 100.6 | 109.7 | 118.8 | 102.2 | 112.4 | 122.7 |
| 17 | 106.7 | 114.4 | 122.1 | 108.3 | 117.4 | 126.6 | 110.2 | 120.4 | 130.6 |
| 18 | 117.2 | 125.6 | 134.0 | 116.0 | 125.3 | 134.6 | 117.1 | 127.1 | 137.2 |
| 19 | 127.6 | 136.7 | 145.8 | 124.1 | 133.7 | 143.2 | 124.0 | 134.0 | 143.9 |
| 20 | 138.0 | 147.7 | 157.5 | 133.1 | 143.2 | 153.2 | 132.4 | 142.5 | 152.6 |
| 21 | 148.3 | 158.7 | 169.1 | 143.2 | 154.0 | 164.8 | 143.8 | 154.3 | 164.8 |
| 22 | 158.6 | 169.6 | 180.6 | 154.0 | 165.7 | 177.3 | 157.7 | 168.7 | 179.7 |
| 23 | 168.9 | 180.4 | 192.0 | 165.1 | 177.6 | 190.1 | 171.4 | 183.0 | 194.6 |
| 24 | 179.0 | 191.2 | 203.3 | 176.3 | 189.7 | 203.2 | 184.6 | 197.0 | 209.3 |
| 25 | 189.1 | 201.8 | 214.5 | 187.7 | 202.0 | 216.3 | 197.1 | 210.4 | 223.7 |
| 26 | 199.1 | 212.4 | 225.7 | 199.0 | 214.2 | 229.4 | 208.9 | 223.2 | 237.5 |
| 27 | 209.1 | 222.9 | 236.8 | 210.2 | 226.2 | 242.3 | 219.8 | 235.2 | 250.6 |
| 28 | 218.8 | 233.3 | 247.8 | 221.0 | 237.8 | 254.6 | 229.7 | 246.2 | 262.7 |
| 29 | 228.5 | 243.6 | 258.7 | 231.4 | 248.8 | 266.3 | 238.7 | 256.2 | 273.7 |
| 30 | 238.0 | 253.8 | 269.6 | 241.0 | 259.0 | 277.0 | 246.6 | 265.0 | 283.4 |
| 31 | 247.4 | 263.9 | 280.5 | 249.9 | 268.1 | 286.4 | 253.4 | 272.4 | 291.5 |
| 32 | 256.5 | 273.9 | 291.3 | 258.1 | 276.3 | 294.5 | 259.7 | 278.9 | 298.0 |
| 33 | 265.5 | 283.8 | 302.2 | 267.1 | 284.6 | 302.1 | 267.3 | 285.8 | 304.2 |
| 34 | 274.3 | 293.6 | 313.0 | 277.7 | 293.9 | 310.1 | 276.5 | 294.0 | 311.5 |
| 35 | 282.8 | 303.3 | 323.8 | 288.6 | 303.4 | 318.3 | 286.5 | 302.9 | 319.2 |
| 36 | 291.0 | 312.8 | 334.6 | 298.1 | 312.0 | 326.0 | 296.6 | 311.9 | 327.2 |
| 37 | 299.0 | 322.3 | 345.5 | 305.3 | 318.8 | 332.3 | 306.7 | 321.0 | 335.3 |
| 38 | 306.7 | 331.6 | 356.4 | 311.0 | 324.2 | 337.3 | 316.5 | 329.6 | 342.7 |
| 39 | 314.1 | 340.8 | 367.4 | 316.1 | 329.0 | 341.9 | 326.1 | 338.1 | 350.1 |
| 40 | 321.1 | 349.8 | 378.5 | 321.0 | 333.6 | 346.3 | 335.6 | 346.6 | 357.5 |

AC: Abdominal circumference
