# Supplementary material for: Comparison of fetal growth patterns from Western India with Intergrowth-21st
Source: PLoS One. 2024 Oct 14;19(10):e0310710. doi: 10.1371/journal.pone.0310710 (PMC11472910; doi:10.1371/journal.pone.0310710)
Supplement: S6 Table — FL: femur length. (DOCX) [file pone.0310710.s006.docx]

**S6 Table: Comparison of REVAMP cohort FL centiles with Intergrowth-21^st^ centiles**

| **FL** | **Intergrowth 21^st^** | | | **REVAMP cohort**  **Total population**  **(655)** | | | **REVAMP cohort**  **Low risk population (106)** | | |
| --- | --- | --- | --- | --- | --- | --- | --- | --- | --- |
|  | **10^th^** | **50^th^** | **90^th^** | **10^th^** | **50^th^** | **90^th^** | **10^th^** | **50^th^** | **90^th^** |
| 14 | 11.2 | 13.1 | 15.1 | 18.2 | 19.6 | 21.1 | 17.8 | 18.8 | 19.9 |
| 15 | 14.3 | 16.3 | 18.3 | 20.2 | 21.8 | 23.4 | 20.2 | 21.5 | 22.8 |
| 16 | 17.4 | 19.5 | 21.5 | 22.1 | 24.0 | 25.8 | 22.5 | 24.1 | 25.7 |
| 17 | 20.4 | 22.5 | 24.7 | 24.1 | 26.1 | 28.2 | 24.4 | 26.4 | 28.4 |
| 18 | 23.4 | 25.5 | 27.7 | 26.0 | 28.3 | 30.7 | 26.1 | 28.6 | 31.0 |
| 19 | 26.2 | 28.5 | 30.7 | 28.2 | 30.8 | 33.4 | 28.1 | 30.9 | 33.6 |
| 20 | 29.0 | 31.3 | 33.6 | 30.6 | 33.5 | 36.4 | 30.5 | 33.5 | 36.6 |
| 21 | 31.7 | 34.1 | 36.4 | 33.3 | 36.4 | 39.5 | 33.2 | 36.5 | 39.8 |
| 22 | 34.4 | 36.7 | 39.1 | 36.0 | 39.3 | 42.6 | 36.0 | 39.5 | 43.0 |
| 23 | 36.9 | 39.4 | 41.8 | 38.8 | 42.2 | 45.6 | 38.8 | 42.6 | 46.4 |
| 24 | 39.4 | 41.9 | 44.4 | 41.5 | 45.0 | 48.5 | 41.6 | 45.7 | 49.7 |
| 25 | 41.8 | 44.4 | 46.9 | 44.2 | 47.8 | 51.4 | 44.4 | 48.7 | 52.9 |
| 26 | 44.1 | 46.7 | 49.3 | 46.9 | 50.5 | 54.1 | 47.2 | 51.6 | 56.0 |
| 27 | 46.4 | 49.0 | 51.7 | 49.5 | 53.1 | 56.7 | 49.8 | 54.3 | 58.8 |
| 28 | 48.6 | 51.3 | 54.0 | 52.0 | 55.5 | 59.1 | 52.4 | 56.8 | 61.3 |
| 29 | 50.6 | 53.4 | 56.2 | 54.3 | 57.8 | 61.3 | 54.7 | 59.0 | 63.4 |
| 30 | 52.6 | 55.5 | 58.4 | 56.5 | 59.9 | 63.3 | 56.9 | 60.9 | 65.0 |
| 31 | 54.6 | 57.5 | 60.5 | 58.5 | 61.7 | 65.0 | 58.8 | 62.5 | 66.2 |
| 32 | 56.4 | 59.4 | 62.5 | 60.4 | 63.5 | 66.5 | 60.7 | 63.9 | 67.0 |
| 33 | 58.2 | 61.3 | 64.4 | 62.3 | 65.2 | 68.0 | 62.7 | 65.3 | 68.0 |
| 34 | 59.8 | 63.1 | 66.3 | 64.2 | 66.9 | 69.6 | 64.6 | 67.0 | 69.3 |
| 35 | 61.4 | 64.8 | 68.1 | 65.8 | 68.5 | 71.1 | 66.4 | 68.6 | 70.7 |
| 36 | 62.9 | 66.4 | 69.9 | 67.2 | 69.8 | 72.3 | 68.5 | 70.6 | 72.6 |
| 37 | 64.3 | 67.9 | 71.6 | 68.2 | 70.8 | 73.4 | 70.8 | 72.8 | 74.7 |
| 38 | 65.6 | 69.4 | 73.2 | 69.1 | 71.7 | 74.3 | 73.2 | 75.0 | 76.9 |
| 39 | 66.9 | 70.8 | 74.7 | 69.9 | 72.6 | 75.2 | 75.5 | 77.3 | 79.0 |
| 40 | 68.0 | 72.1 | 76.2 | 18.2 | 19.6 | 21.1 | 17.8 | 18.8 | 19.9 |

FL: Femur length
